# Demonstrating Intertumoural Differences in Vascular-Metabolic Phenotype with Dynamic Contrast-Enhanced CT-PET

**DOI:** 10.1155/2011/679473

**Published:** 2011-04-26

**Authors:** K. A. Miles, R. E. Williams, D. Yu, M. R. Griffiths

**Affiliations:** ^1^Brighton and Sussex Medical School, University of Sussex, Brighton BN1 9RH, UK; ^2^Clinical Imaging Sciences Centre, Brighton and Sussex Medical School, University of Sussex, Falmer, Brighton BN1 2PB, UK; ^3^Queensland PET Service, Royal Brisbane and Women's Hospital, Brisbane, QLD 4029, Australia

## Abstract

*Purpose*. To assess whether the differences in vascular-metabolic relationships between lymphoma masses and colorectal liver metastases predicted from previous histopathological studies can be demonstrated by dynamic contrast-enhanced CT (DCE-CT) combined with fluorodeoxyglucose positron emission tomography (FDG-PET). *Methods*. DCE-CT and FDG-PET studies were drawn from an imaging archive for patients with either lymphoma masses (*n* = 11) or hepatic metastases from colorectal cancer (CRM: *n* = 12). Tumour vascularity was assessed using DCE-CT measurements of perfusion. Tumour glucose metabolism was expressed as the mean FDG Standardised Uptake Value (SUV_FDG_). The relationship between metabolism and vascularity in each group was assessed from SUV_FDG_ /perfusion ratios and Pearson correlation coefficients. *Results*. An SUV_FDG_ threshold of 3.0 was used to designate lymphoma masses as active (AL, *n* = 6) or inactive lymphoma (IL, *n* = 5). Tumour perfusion was significantly higher in AL (0.65 mL/min/mL) than CRM (0.37 mL/min/mL: *P* = .031) despite similar SUV_FDG_ (5.05 and 5.33, resp.). AL demonstrated higher perfusion values than IL (0.24 mL/min/mL: *P* = .006). SUV_FDG_/perfusion was significantly higher in CRM (15.3 min) than IL (4.2 min, *P* < .01). There was no correlation between SUV_FDG_ and perfusion for any patient group.

## 1. Introduction

The use of multiparametric imaging to study the relationship between vascularisation and metabolism of tumours is emerging as a topic of interest in oncological imaging [1]. Increased vascularity and increased glucose metabolism are both important factors in the growth and metastasis of many tumours [2–8]. On the other hand, poor vascularisation may lead to hypoxia which is known to contribute to resistance to radiotherapy and chemotherapy. The degree of vascularisation and metabolism represent the ultimate phenotypic expression of a range of genetic processes occurring within tumour cells. Imaging the relationship between metabolism and vascularity can therefore define tumour phenotypes of potential prognostic significance [9].

Previous studies evaluating the relationship between tumour metabolism and vascularity have combined fluoro-18-deoxyglucose positron emission tomography (FDG-PET) images of metabolism with a range of techniques that depict tumour vascularity including O-15 water PET, dynamic contrast-enhanced magnetic resonance and dynamic contrast-enhanced computed tomography (DCE-CT) [1]. Of these techniques, DCE-CT is most readily performed on integrated PET-CT systems remote from a cyclotron. DCE-CT is increasingly used to evaluate tumour vascularity on account of the technique's ability to provide indirect in vivo assessments of tumour angiogenesis and oxygenation [10–17]. Tumour perfusion values derived from kinetic analysis of DCE-CT have been validated against a range of reference methods in animals and man [18–21]. Studies that have compared FDG-PET and DCE-CT in lung, head and neck, and breast cancers have shown the ability of combined measurements to demonstrate variations in the relationship between tumour blood flow and metabolism within a single tumour type [22–26]. Uncoupling of tumour perfusion and metabolism has been identified as a feature of pulmonary metastases and also larger, more advanced nonsmall cell lung cancers and cancers of the head and neck [22, 23]. More recently, a preliminary study has suggested differences in vascular-metabolic ratios between grade 2 and grade 3 breast cancers [25]. However, to date, studies of tumour metabolism and vascularity have been undertaken in single tumour types. There have been no studies utilising FDG-PET with DCE-CT to evaluate differences in vascular-metabolic phenotype between different tumours. 

Lymphomas are known to exhibit a range of biological characteristics that differ from those observed in other tumour types. In particular, immunohistopathological studies have suggested that Hodgkin's and Non-Hodgkin's lymphomas may be less angiogenic and hypoxically driven than most solid tumours [26, 27]. Thus, it can be anticipated that lymphomas will be unlikely to exhibit hypoxic stimulation of glucose metabolism seen as low vascularity on DCE-CT and high glucose metabolism on FDG-PET. On the other hand, similar studies in colorectal cancer liver metastases have shown large amounts of hypoxic cells but with no correlation between the hypoxia marker pimonidazole and the expression of the GLUT-1 glucose transporter [28]. Hence, colorectal metastases would be expected to show relatively low vascularity (reflecting hypoxia) and no relationship between vascularity and glucose metabolism. The purpose of this study was to undertake a preliminary comparison of tumour metabolism and vascularity in lymphoma masses and colorectal liver metastases to assess whether the differences in vascular-metabolic phenotype predicted from previous histopathological studies can be demonstrated by combining DCE-CT with FDG-PET. The ability to demonstrate such intratumoural differences would provide further validation of the technique as a means to characterise the biological behaviour of tumours.

## 2. Methods and Materials

### 2.1. Patients

The study comprised a retrospective review of image data for patients with lymphoma and colorectal cancer drawn from an imaging archive acquired between 1999 and 2002 as part of a research program evaluating angiogenesis and glucose metabolism in a range of tumour types. Patients had been recruited sequentially, and all had undergone FDG-PET and CT as part of their clinical care with a single-location DCE-CT added to the conventional diagnostic CT. PET and CT examinations had been performed within 48 hours using separate imaging systems. This research program had been approved by the local ethics committee, and informed written consent had been obtained from all patients. 

Image data was available for 19 studies in 18 patients with biopsy-proven lymphoma. Eight studies acquired after lymphoma therapy were rejected for having no measurable disease at the anatomical location chosen for DCE-CT. Of the remaining patients, one had been studied before and after treatment giving 11 available studies in 10 patients (7 male, 3 female, median age: 58.1 years, range: 17–83). The lymphoma subtype had been recorded in the archive for only 5 patients (2 Hodgkin's disease, 3 non-Hodgkin's lymphoma). Lymphoma studies were classified as active or inactive disease on the basis of the FDG uptake within the lesion on the slice corresponding to the DCE-CT acquisition. A Standardised Uptake Value for FDG (SUV_FDG_) of greater than 3.0 was considered to indicate active disease, a threshold value which has previously been shown to predict a significantly poorer progression-free survival [29].

Image data was also available for 40 patients with known or suspected recurrent colorectal cancer. Five cases were excluded due to incomplete image data sets, and a further 23 were excluded due to no measurable disease at the anatomical location chosen for DCE-CT. Thus, 12 patients were available for analysis, all of which had an FDG-avid focal liver lesion in the anatomical slice corresponding to that chosen for DCE-CT. One patient had been studied before and after treatment with 5-fluorouracil and levamisole. Only the pretreatment data was used in this study.

### 2.2. Positron Emission Tomography

A standard PET imaging technique had been used for all patients in the archive. Following a 6-hour fast and confirmation of serum glucose levels below 10 mmoL/L, whole-body PET images of patients had been acquired 60 minutes after intravenous administration of 120–200 MBq FDG using a GE QUEST dedicated PET scanner (GE Medical Systems, Milwaukee, WI). Transmission scanning had been performed and attenuation corrected images (128x128 pixel, slice thickness of 4 mm) produced by iterative reconstruction using the ordered subset expectation maximization (OSEM) algorithm. Values for FDG uptake within the tumour, expressed as the mean Standardised Uptake Value (SUV), had been determined from the PET image at the anatomical level corresponding to the DCE-CT acquisition and recorded within the image archive.

### 2.3. CT Image Acquisition

The DCE-CT study had been incorporated into the patients' conventional CT examination as follows: (1) a series of unenhanced images were acquired to determine the appropriate anatomical level for (2) a dynamic single-location contrast-enhanced sequence followed by (3) a conventional diagnostic portal phase examination of the chest, abdomen, and pelvis performed using an additional 100 mL bolus of contrast material at least 5 minutes after the single-location acquisition (CT Twin: Elscint, Haifa, Israel). Prior to imaging, contrast medium (Ioscan 10 mL, sodium diatrizoate 3.705 g; Iotech, Rosebery, Australia) had been given via mouth following a 4-hour fast.

The single-location DCE-CT sequence of images had been acquired at the anatomical level containing the largest transverse dimension of any focal mass lesion identified on the unenhanced images. 50 mL of conventional contrast material (Iopamidol, Bracco, Milan) with an iodine concentration of 370 mg/mL had been administered intravenously at 7 mL/sec through an 18 G cannula. Patients had been instructed not to take deep breaths but to breathe quietly for the entire duration of the sequence. Data acquisition started at the time of contrast material injection. For all patients apart from 3 with lymphoma, 12 1-second images with a slice thickness of 10 mm (120 kVp, 300 mAs) had been obtained using a cycle time of 3 seconds. For the remaining 3 patients, 60 1-second images had been acquired with a cycle time of 1 sec using the same tube voltage but a reduced tube current. The CT images stored in the archive in Digital Imaging and Communications in Medicine (DICOM) format were reanalysed for this study.

### 2.4. CT Image Analysis

CT analysis was performed by, or under the supervision of, an operator with 18 years of experience in perfusion CT imaging (KM) blinded to the PET findings. For each patient, a region of interest (ROI) encompassing the lymphoma mass or liver lesion was created from the precontrast CT image displayed with a constant window width and level (300 HU and 0 HU, resp.). Further ROIs were created within the aorta and, for liver lesions, within the spleen. The changes in the X-ray attenuation within each ROI following administration of contrast material were displayed as time-attenuation curves (TACs). For liver lesions, the splenic TAC was used to determine the time of peak splenic enhancement identifying the end of the arterial phase. Only arterial phase images (i.e., those acquired up to the time of peak splenic enhancement) were included in the analysis to reflect the fact that hepatic tumours receive their blood supply predominantly from the hepatic arterial system [30]. Individual images subject to excessive respiratory motion were eliminated from analysis. TACs from the three studies acquired with a cycle time of 1 second underwent 3 point temporal smooth to ensure equivalence with the other studies acquired with a cycle time of 3 seconds. 

Tumour perfusion was calculated as described by Miles [31] using the following equation:

(1) $$\begin{array}{cc} & \text{Tumour}\;\text{perfusion}\;\left(\text{mL}/\min\;/\text{mL}\right)\\ & \;=\frac{\text{Maximal}\;\text{rate}\;\text{of}\;\text{tumour}\;\text{enhancement}\;\left(\text{HU}/\min\;\right)}{\text{Peak}\;\text{aortic}\;\text{enhancement}\;\left(\text{HU}\right)}. \end{array}$$

This approach to measuring tumour perfusion has been validated against thermal clearance and PET using the steady-state oxygen-15 labelled carbon dioxide method [21].

### 2.5. Definition of Vascular-Metabolic Phenotypes

Four vascular-metabolic phenotypes were defined using the following metabolic-vascular domains categorised by threshold values for SUV_FDG_ and perfusion: (a) low metabolism/low perfusion, (b) low metabolism/high perfusion, (c) high metabolism/high perfusion, and (d) high metabolism/low perfusion. The threshold value for SUV_FDG_ was 3.0 [29]. As no equivalent perfusion threshold has been determined previously, the median perfusion value of all included patients was used.

### 2.6. Statistical Analysis

The median value and interquartile range were calculated for SUV_FDG_, perfusion, and SUV_FDG_/perfusion. Differences in these parameters between patient groups (i) active lymphoma (AL), (ii) inactive lymphoma (IL), and (iii) colorectal cancer liver metastases (CRM) were tested for significance using the Mann-Whitney test for nonparametric data. Pearson correlation coefficients were estimated between FDG_SUV_ and perfusion for each group. The distribution of patients within the four vascular-metabolic phenotypes was tested for significance using Fisher's exact test for a 2 × 2 table. For all statistical tests, *P*-values of .05 or less were considered significant.

## 3. Results

**Figure 1:**
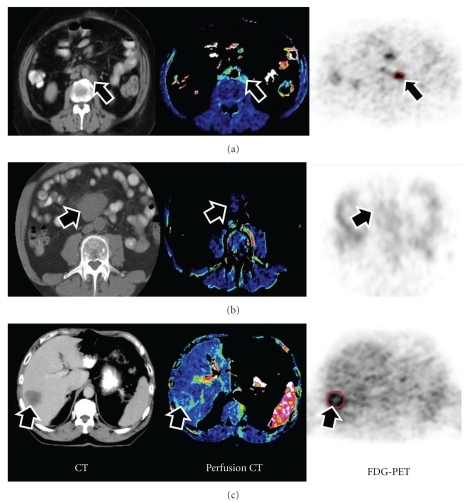
Conventional CT (left column), perfusion CT (middle column), and FDG-PET (right column) images of patients with (a) active lymphoma, (b) inactive lymphoma, and (c) colorectal liver metastases. Arrows indicate the tumour masses. Active lymphoma demonstrated high blood flow and high FDG uptake, inactive lymphoma, low blood flow and low FDG uptake, whilst the colorectal liver metastasis demonstrates low blood flow and high glucose metabolism.

Figure 1 comprises examples of conventional CT, perfusion CT, and FDG-PET images from patients with active lymphoma, inactive lymphoma, and colorectal liver metastases. Tables 1a and 1b list the demographics and values of perfusion and FDG_SUV_ for each patient with active lymphoma, inactive lymphoma, and colorectal liver metastases. Pairwise comparisons of patient groups showed tumour perfusion was significantly higher in AL (0.65 mL/min/mL) than CRM (0.37 mL/min/mL: *P* = .031) despite similar SUV_FDG_ (5.05 and 5.33, resp.). Active lymphoma masses demonstrated higher perfusion values than inactive lymphoma (0.24 mL/min/mL: *P* = .006). The SUV_FDG_ /perfusion ratio was significantly higher in CRM (15.3 min) than IL (4.2 min, *P* < .01). Refer to Table 2 and Figure 3. 

**Figure 2:**
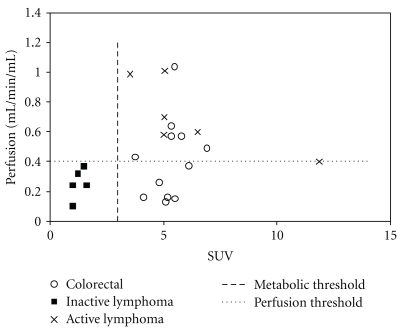
Relationships between FDG Standardised Uptake Value (SUV) and blood flow for active lymphoma, inactive lymphoma, and colorectal liver metastases.

**Figure 3:**
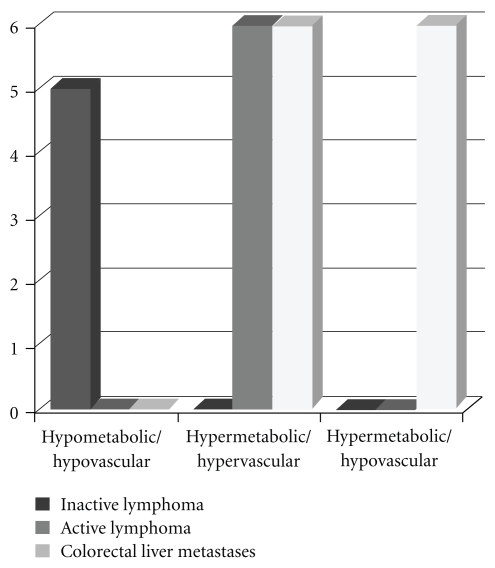
Prevalence of tumour type in each metabolic-vascular domain (hypometabolic: SUV <3, hypovascular: perfusion <0.4 mL/min/mL, *P* < .00005, Fisher's exact test).

Figure 2 displays the relationships between FDG_SUV_ and perfusion for the three patient groups. Linear regression analysis showed no statistically significant correlation between these two parameters for any patient group. The distribution of cases within the metabolic-vascular domains based on thresholds of ≥3.0 for SUV_FDG_ and the median perfusion value of all cases (≥0.4 mL/min/mL) was significantly non-random (*P* < .00005), All cases of IL fell within the low metabolism/low perfusion domain whereas all cases of AL exhibited high metabolism and high perfusion. Colorectal liver metastases were evenly distributed between high metabolism/high perfusion and high metabolism/low perfusion domains. No cases were classified as low metabolism/high perfusion.

## 4. Discussion

Our results confirm that combined FDG-PET and DCE-CT has the potential to evaluate vascular-metabolic relationships in lymphoma and colorectal liver metastases. The differences between active lymphoma and colorectal liver metastases demonstrated by this imaging approach are consistent with previously reported histopathological studies. 

Our main finding was that, although active lymphoma and colorectal liver metastases exhibited similar levels of metabolism, there were significant differences in perfusion between these tumour types. In view of the previously demonstrated correlation between CT perfusion measurements and tumour oxygenation [16], our main finding corresponds to previous immunohistochemical studies indicating that hypoxia is uncommon in lymphoma but is a prominent feature in colorectal liver metastases [26–28]. However, it is also interesting that colorectal liver metastases with low perfusion tended to exhibit SUV_FDG_ no higher than either colorectal liver metastases with high perfusion or active lymphoma. This latter finding is in contrast to previous studies in nonsmall lung cancer and head and neck tumours which have shown that low levels of tumour vascularity are often associated with particularly high levels of glucose metabolism [22, 23]. Hypoxia is a recognised stimulus for increased glucose metabolism, which can be considered as an adaptive response to hypoxia [32]. Our finding of comparable metabolism in high and low perfusion in colorectal liver metastases suggests a failure of adaptation to hypoxia in this tumour type. This finding is also consistent with published results of histochemical analysis of colorectal liver metastases which found no correlation between the hypoxia marker pimonidazole and the expression of the GLUT-1 glucose transporter [28].

To our knowledge, this is the first study to use FDG-PET and DCE-CT to compare the relationship between vascularity and metabolism in different tumour types. However, the relationships between perfusion and metabolism in lymphoma and colorectal liver metastases have been assessed previously in separate studies [33, 34]. Dimitrakopoulou-Strauss et al. used PET measurements of SUV for oxygen-15 water (SUV_water_) and FDG to assess tumour perfusion and metabolism in patients with malignant lymphomas before and after treatment, and they reported a nonlinear correlation between these two parameters [33]. This contrasts with our study in that no correlation between perfusion and metabolism was observed in patients with either active or inactive lymphoma. Nevertheless, 5 minute after injection, SUV_water_ measurements may not be directly equivalent to absolute measurements of perfusion. Amongst our patients, all cases of inactive lymphoma had perfusion values of less than 0.4 mL/min/mL. This threshold value is higher than the 0.2 mL/min/mL value previously proposed by Dugdale et al. [35]. Unlike our study, the series of Dugdale et al. did not classify disease activity on the basis of FDG-PET. We found no overlap in perfusion values between active and inactive cases suggesting that, for lymphoma masses, perfusion and SUV_FDG_ values may provide similar information. Larger trials would be needed to confirm this finding. 

Fukuda et al. used oxygen-15 water measurements and FDG-PET to study perfusion and metabolism in liver tumours including six colorectal liver metastases [34]. The median values for perfusion and FDG_SUV_ in that study (0.32 mL/min/mL and 4.6, resp.) were very similar to those in our patient group (0.37 mL/min/mL and 5.3, resp.). Both studies also found no correlation between tumour metabolism and perfusion in colorectal liver metastases, implying that these parameters provide different information about the biological status of such tumours and highlighting the potential for combined DCE-CT/FDG-PET to provide distinctive information about tumour biology. 

The two previous studies of perfusion and metabolism in lymphoma and colorectal liver metastases both used oxygen-15 water PET rather than DCE-CT to assess tumour vascularity. There are important differences between these two techniques. Oxygen-15 water is a diffusible tracer that will more closely reflect the delivery of nutrients to tumour tissue. The presence of arteriovenous shunts will not affect oxygen-15 water measurements of perfusion but will contribute to perfusion values obtained with DCE-CT. Although not contributing to the delivery of nutrients to tumour tissue, arteriovenous shunts are an important pathological feature that distinguishes tumour vascularisation from the vascularisation of normal tissues. Thus, by capturing the effects of arteriovenous shunting, DCE-CT measurements of perfusion will depict an aspect of tumour vascularity not detected by oxygen-15 water PET. The use of DCE-CT can also avoid some of the artefacts that may occur with oxygen-15 water PET such as spill over of counts from adjacent vascular structure which may be an important consideration for assessing lymphoma masses which are frequently located close to the aorta and mesenteric vessels. Notwithstanding these theoretical differences, our DCE-CT measurements of perfusion with CRM were comparable to those reported by Naumann et al. and recent comparative study of oxygen-15 water PET and DCE-CT measurements of perfusion in solid tumours found a good correlation between the two techniques [29]. 

Our study identified threshold values for SUV_FDG_ (≥3.0) and perfusion (≥0.4 mL/min/mL) defining vascular-metabolic domains that effectively distinguished the three patients groups (active lymphoma, inactive lymphoma, and colorectal liver metastases). Increased glucose metabolism is a fundamental property of tumour cells whereas the new vessels resulting from angiogenesis are predominantly derived from the surrounding native vascular system. Thus, identification of specific vascular-metabolic domains may be able to classify tumours on the basis of the biological behaviour of the tumour itself and the host's response to the tumour. Clinical studies in a range of tumour types have shown variable and inconsistent relationships between tumour perfusion and metabolism [1, 22–24, 33, 34, 36–40]. High metabolism with low vascularity has previously been demonstrated in larger, more advanced tumours, [22, 36] and adjacent to areas of necrosis [27, 28]. Furthermore, mismatching of tumour perfusion and metabolism may also be induced by cancer therapy, particularly drugs that target the tumour vasculature [41, 42]. Our study has shown that perfusion-metabolic mismatch is also common in colorectal liver metastases.

An important limitation of our study is the retrospective analysis of a relative small number of patients drawn from an imaging archive with incomplete clinical data. Furthermore, improvements of DCE-CT technique and PET technology have occurred since the creation of the archive. For example, a lower tube-voltage (80–100 kVp) is now recommended for DCE-CT than that widely adopted at the time our cases were acquired, and the increased axial field of view of current CT systems allows study of greater tissue volume with the potential to reduce the impact of breathing artefacts. Nevertheless, the increasing use of contrast agent during clinical PET-CT [43, 44] will allow DCE-CT and FDG-PET to be combined in a single examination and so facilitate the larger prospective studies that are needed to confirm our findings and to evaluate the potential for combined parameters of perfusion and FDG uptake to provide prognostic information. 

The potential clinical implications of our study are different for lymphoma and colorectal liver metastases. For lymphoma, if the apparent equivalence of FDG-PET and DCE-CT for assessing disease activity is confirmed in larger trials, it is possible that DCE-CT could become a feasible technique for a preliminary assessment of residual lymphoma masses on completion of chemotherapy. Perfusion values remaining above 0.4 mL/min/mL could potentially be used to select patients for consolidation therapy. On the other hand, the inability of DCE-CT to image multiple tumour sites would prevent the withholding of consolidation therapy on the basis of a low perfusion value in a single target lymphoma mass because residual disease activity could be present in other lesions not assessed by DCE-CT. For colorectal liver metastases, it is feasible that combined FDG-PET/DCE-CT could be used to classify liver lesions in a novel way on the basis of their vascular-metabolic domain. Metastases exhibiting high metabolism/high perfusion may have a different prognosis from those with high metabolism/low perfusion. This potential prognostic value is suggested by two previous studies that have used either FDG-PET or DCE-CT in separate series of patients with hepatic metastases. These studies suggest that high glucose metabolism and low perfusion in CRM are both associated with reduced survival [45]. However, studies that combine imaging with clinical followup are needed to confirm the potential prognostic value of this combined technique. 

In summary, this preliminary study has demonstrated the potential for combined FDG-PET and DCE-CT to demonstrate differences in vascular-metabolic relationships in lymphoma and colorectal liver metastases. The differences shown are consistent with the findings of previous histopathological studies. The higher perfusion values found in active lymphoma compared to colorectal liver metastases are consistent with a higher incidence of hypoxia in colorectal liver metastases. However, this reduction in blood flow was not associated with a greater SUV_FDG_, suggesting poor adaptation to hypoxia in this tumour type.

## Figures and Tables

**Table:** (a) Demographics and imaging results for patients with lymphoma. (*N* = non-Hodgkin's lymphoma, *H* = Hodgkin's disease).

Tumour type	Age (years)/sex	FDG SUV	Blood flow (mL/min/mL)
Active lymphoma^*N*^	65/M	3.5	0.99
Active lymphoma	71/F	5.0	0.58
Active lymphoma	40/F	5.0	0.7
Active lymphoma^*H*^	62/M	5.1	1.01
Active lymphoma^*N*^	56/M	6.5	0.6
Active lymphoma	83/M	11.9	0.4

Median (Interquartile range)	64 (57–69)	5.0 (5.0–6.1)	0.65 (0.59–0.92)

Inactive lymphoma^*N*^	40/F	1	0.24
Inactive lymphoma	51/M	1	0.1
Inactive lymphoma	58/M	1.2	0.32
Inactive lymphoma^*H*^	17/F	1.5	0.37
Inactive lymphoma^*N*^	77/M	1.6	0.24

Median (Interquartile range)	51 (40–58)	1.2 (1.0–1.5)	0.24 (0.24–0.32)

**Table:** (b) Demographics and imaging results for patients with colorectal liver metastases.

Tumour type	Age/sex	FDG SUV	Blood flow (mL/min/mL)
Colorectal cancer	73/F	5.5	0.15
Colorectal cancer	49/M	5.5	1.04
Colorectal cancer	58/F	3.8	0.43
Colorectal cancer	52/F	5.3	0.57
Colorectal cancer	64/M	6.9	0.49
Colorectal cancer	73/M	4.8	0.26
Colorectal cancer	75/F	4.1	0.16
Colorectal cancer	56/F	5.8	0.57
Colorectal cancer	50/M	5.1	0.13
Colorectal cancer	72/M	5.2	0.16
Colorectal cancer	65/M	5.3	0.64
Colorectal cancer	87/F	6.1	0.37

Median (Interquartile range)	65 (55–73)	5.3 (5.0–5.6)	0.37 (0.16–0.57)

**Table 2:** Prevalence of tumour type in each metabolic-vascularity domain (hypometabolic: SUV <3, hypovascular: perfusion <0.4 mL/min/mL, *P* < .00005, Fisher's exact test).

	Inactive lymphoma	Active lymphoma	Colorectal liver metastases
Hypometabolic/ hypovascular	5	0	0
Hypermetabolic/ hypervascular	0	6	6
Hypermetabolic/ hypovascular	0	0	6
